# Development and Validation of Machine Learning Models for Real-Time Mortality Prediction in Critically Ill Patients With Sepsis-Associated Acute Kidney Injury

**DOI:** 10.3389/fmed.2022.853102

**Published:** 2022-06-15

**Authors:** Xiao-Qin Luo, Ping Yan, Shao-Bin Duan, Yi-Xin Kang, Ying-Hao Deng, Qian Liu, Ting Wu, Xi Wu

**Affiliations:** Department of Nephrology, Hunan Key Laboratory of Kidney Disease and Blood Purification, The Second Xiangya Hospital of Central South University, Changsha, China

**Keywords:** sepsis, acute kidney injury, mortality, machine learning, critical care

## Abstract

**Background:**

Sepsis-associated acute kidney injury (SA-AKI) is common in critically ill patients, which is associated with significantly increased mortality. Existing mortality prediction tools showed insufficient predictive power or failed to reflect patients' dynamic clinical evolution. Therefore, the study aimed to develop and validate machine learning-based models for real-time mortality prediction in critically ill patients with SA-AKI.

**Methods:**

The multi-center retrospective study included patients from two distinct databases. A total of 12,132 SA-AKI patients from the Medical Information Mart for Intensive Care IV (MIMIC-IV) were randomly allocated to the training, validation, and internal test sets. An additional 3,741 patients from the eICU Collaborative Research Database (eICU-CRD) served as an external test set. For every 12 h during the ICU stays, the state-of-the-art eXtreme Gradient Boosting (XGBoost) algorithm was used to predict the risk of in-hospital death in the following 48, 72, and 120 h and in the first 28 days after ICU admission. Area under the receiver operating characteristic curves (AUCs) were calculated to evaluate the models' performance.

**Results:**

The XGBoost models, based on routine clinical variables updated every 12 h, showed better performance in mortality prediction than the SOFA score and SAPS-II. The AUCs of the XGBoost models for mortality over different time periods ranged from 0.848 to 0.804 in the internal test set and from 0.818 to 0.748 in the external test set. The shapley additive explanation method provided interpretability for the XGBoost models, which improved the understanding of the association between the predictor variables and future mortality.

**Conclusions:**

The interpretable machine learning XGBoost models showed promising performance in real-time mortality prediction in critically ill patients with SA-AKI, which are useful tools for early identification of high-risk patients and timely clinical interventions.

## Introduction

Sepsis is life-threatening organ dysfunction due to a dysregulated host response to infection. It is a major cause of health loss worldwide (1, 2). Acute kidney injury (AKI), characterized by an abrupt increase in serum creatinine (SCr) or decrease in urine output, is a common complication of critical illness (3–5). AKI has been shown to be more frequent, less likely to resolve, and associated with higher mortality in critically ill patients with sepsis than in those without (6). Considering the critical condition of patients with sepsis-associated AKI (SA-AKI), the accurate prediction of their outcomes is a topic of interest.

Studies have shown that widely-used severity scores, such as the Simplified Acute Physiology Score II (SAPS-II) and the Sequential Organ Failure Assessment (SOFA) score, exhibit insufficient power for outcome prediction in SA-AKI patients (7, 8). A few prediction models for mortality in patients with SA-AKI have been established (7, 8). However, they were limited to small sample size or inadequate predictive performance. In addition, the models incorporated static measurements at single time points, typically in the early period after intensive care unit (ICU) admission, and failed to reflect patients' dynamic clinical evolution. There is still a lack of feasible ways to assess the real-time risk of death and guide individualized treatment decisions in critically ill patients with SA-AKI.

The rapid development in big data analytics and machine learning techniques, along with the data-rich environment in ICU settings, provide unprecedented opportunities to establish novel mortality prediction tools in SA-AKI patients (9–11). Advanced machine learning methods are adept at handling high-order interactions and fitting complex non-linear relationships, which can be used to integrate large amounts of data from electronic health records (EHRs). The application of data-driven analytics by machine learning has shown promise to improve predictive performance in medical fields (12–15).

The study aimed to develop and validate machine learning-based models for real-time mortality prediction in critically ill patients with SA-AKI, in an attempt to provide useful tools for early prognostic assessment and clinical decision-making.

## Methods

### Source of Data

Data were obtained from the Medical Information Mart for Intensive Care IV (MIMIC-IV) v1.0 and the eICU Collaborative Research Database (eICU-CRD) v2.0 (16–19). The MIMIC-IV is a large and publicly available database containing records from patients admitted to the ICUs of the Beth Israel Deaconess Medical Center from 2008 to 2019. The eICU-CRD is a multi-center telehealth database including data from more than 200,000 admissions to 335 ICUs at 208 hospitals across the United States between 2014 and 2015. The study was an analysis of the third-party databases with pre-existing institutional review board approval and all protected patient information de-identified. One of the authors has completed the Collaborative Institutional Training Initiative course and can access the databases (certification number 40010711).

### Study Population

The study included adult patients with sepsis who developed AKI within 48 h after ICU admission. In the MIMIC-IV, sepsis was diagnosed based on the Sepsis-3 criteria, including suspected infection and a SOFA score ≥ 2 (1). We identified patients with suspected infection (antibiotics administration concomitant with body fluid cultures) during the first 24 h after ICU admission and calculated SOFA scores using data from the same period (20). In the eICU-CRD, sepsis was identified according to the admission diagnosis recorded on the Acute Physiology and Chronic Health Evaluation IV dataset (21). AKI was defined based on the 2012 Kidney Disease: Improving Global Outcomes Clinical Practice Guideline, using both SCr and urine output criteria (3). Baseline SCr was defined as the minimum SCr value in the 7 days prior to ICU admission, or the first SCr value after ICU admission if no pre-admission SCr was available (22, 23). If the patient had multiple ICU admissions during a hospital stay, only the first ICU stay was included in the analysis to ensure the independence of the data. Patients with age < 18 years old, end-stage renal disease (identified by diagnosis codes), and ICU stay < 48 hours were excluded.

### Outcomes and Predictor Variables

The primary outcome was in-hospital mortality within 28 days after ICU admission, censored at hospital discharge or 28 days, whichever occurred first. Each patient's ICU stay within 28 days was separated into 12-hour windows, which were labeled as “death” or “survival”. Specifically, to predict mortality in the next 48, 72, and 120 h, the time windows in the corresponding hours before death were labeled as “death” and the remaining as “survival”. To predict mortality in the first 28 days after ICU admission, all time windows were labeled as “death” in patients who died and “survival” in patients who survived. The final objective of the model was to predict the correct label for each time window. Additionally, the secondary outcomes were ICU length of stay, hospital length of stay and use of renal replacement therapy (RRT) within the first 28 days.

The predictor variables within each time window contained four static features (age, sex, ethnicity, and baseline SCr) and sets of dynamic features including hours from ICU admission, vital signs, laboratory values, and interventions. The list of all predictor variables included for modeling is provided in Table 1. For dynamic features, their values were time-varying and updated on a 12-hour basis. We used the mean value of variables measured multiple times and the lowest Glasgow Coma Scale (GCS) score in each time window. For variables with no recorded measurements during the 12-hour windows, their values were carried forward from the most recent measurements.

**Table 1:** List of the predictor variables.

	**Variables**	**Type**
Demographics	age, sex, ethnicity	static
Length of stay	hours from admission	dynamic
Vital signs	systolic blood pressure, diastolic blood pressure, heart rate, respiratory rate, body temperature, oxygen saturation, glasgow coma scale score, urine output	dynamic
Laboratory data	baseline serum creatinine	static
	hemoglobin, white blood cells, platelets, serum total bilirubin, serum albumin, serum creatinine, blood urea nitrogen, arterial pH, partial pressure of arterial oxygen, partial pressure of arterial carbon dioxide, serum sodium, serum potassium, serum chloride, serum bicarbonate, lactate, international normalized ratio, partial thromboplastin time	dynamic
Interventions	mechanical ventilation, vasopressors, renal replacement therapy, loop diuretics	dynamic

### Statistical Analysis

Statistical analyses were performed using R 4.1.2 (https://cran.r-project.org). Continuous variables were presented as medians with interquartile ranges and categorical variables were presented as numbers with percentages. The schematic diagram of methods is shown in Supplementary Figure S1. We divided the study population in the MIMIC-IV into the training (50%), validation (30%), and internal test (20%) sets, randomized at the patient level to ensure that each patient was allocated to only a subset. We used the cohort of SA-AKI patients in the eICU-CRD as an external test set. In the training set, the eXtreme Gradient Boosting (XGBoost) algorithm was used to establish mortality prediction models with all predictor variables input. XGBoost, a scalable end-to-end tree boosting system, is an optimized implementation of the gradient boosting framework designed to be highly efficient, flexible, and portable (24). During the training process, it generates a series of decision trees, each of which is generated based on the previous one to decrease the gradient of the loss function. After that, a prediction model composed of multiple decision trees is obtained. The XGBoost algorithm can handle missing values by adding a default direction for them in each tree node and learning the optimal direction from the data. Therefore, missing values were directly input into the XGBoost models as not available values. Supplementary Table S1 provides the percentages of missing values in the predictor variables. For machine learning approaches, hyperparameter tuning is required to fit the complex relationship in the data and avoid overfitting. The hyperparameters in the XGBoost models (learning rate, minimum sum of instance weight, maximum tree depth, and minimum loss reduction) and max number of boosting iterations were optimized on the validation set to achieve the maximum area under the receiver operating characteristic curves (AUCs). The *xgboost* package was used for XGBoost modeling. Details on the functions and tuning parameters used for the XGBoost algorithm can be found in Supplementary Table S2. More details about the XGBoost algorithm can be found at XGBoost Documentation (https://xgboost.readthedocs.io/).

The performance of the prediction models was assessed on the internal and the external test sets. AUC was selected as the primary evaluation metric. Other metrics included sensitivity, specificity, and accuracy. We reported the metrics under multiple cutoff values, based on the local maximas of the receiver operating characteristic curves. We compared the performance of the XGBoost models with traditional risk scores, including the SOFA score (25) and SAPS-II (26). We did not calculate the risk scores in each 12-hour window for patients in the eICU-CRD because some required variables were unavailable.

The XGBoost algorithm provides the importance of features in predicting the outcome. We used the gain as the measure, representing the fractional contribution of each feature to the model output based on the total gain of this feature's splits. To explore the interpretability of the XGBoost models, we used the Shapley Additive exPlanations (SHAP) method (27), which provides consistent and locally accurate attribution values for each feature. The influence of the predictor variables on the outcome can be explained by the summing effects of variable attributions in calculating the output risk for each observation.

In sensitivity analysis, we applied other frequently used machine learning algorithms such as random forest and support vector machine to our dataset for comparison (28, 29). Additionally, we assessed the performance of the SOFA score, SAPS-II and XGBoost model using data gathered in the early period after ICU admission, i.e., the first 12 h, in predicting in-hospital mortality in the first 28 days.

## Results

### Baseline Characteristics and Outcomes

**Figure 1:**
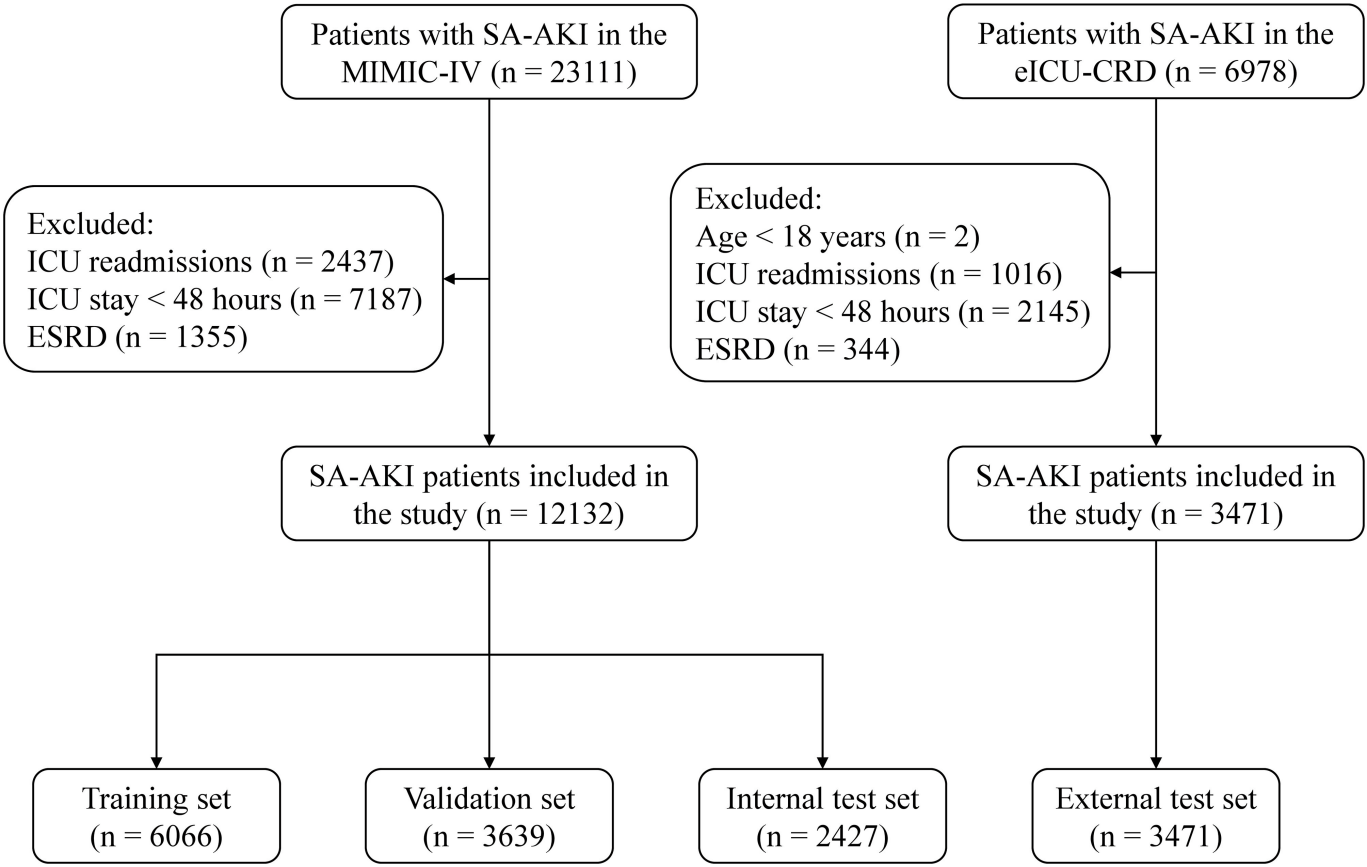
Study flow diagram. SA-AKI, sepsis-associated acute kidney injury; ICU, intensive care unit; ESRD, end-stage renal disease.

A total of 15,603 critically ill patients with SA-AKI were included in our study, with 6,066 in the training set, 3,639 in the validation set, 2,427 in the internal test set, and 3,471 in the external test set (Figure 1). Baseline characteristics and outcomes of the study population in each dataset are shown in Table 2 and Supplementary Table S3. In the MIMIC-IV, 56.6% of SA-AKI patients were diagnosed by urine output criteria, 9.2% by SCr criteria, and 34.2% by both criteria. In the eICU-CRD, the proportions of SA-AKI patients meeting urine output criteria, SCr criteria, and both criteria were 38.5, 40.9, and 20.5%, respectively. The overall in-hospital mortality within 28 days was 18.6% in the training set, 17.0% in the validation set, 18.3% in the internal test set, and 22.7% in the external test set. For each 12 h window of the ICU stays, the number of in-hospital deaths in the first 28 days is shown in Supplementary Table S4. Distribution of the predictor variables within each 12-hour window of the ICU stays is shown in Supplementary Table S5.

**Table 2:** Baseline characteristics and outcomes of SA-AKI patients in the training, validation and internal test sets.

**Variables**	**Training set (*n* = 6,066)**	**Validation set (*n* = 3,639)**	**Internal test set (*n* = 2,427)**
Age (year)	69 (58–79)	70 (59–80)	69 (59–80)
Sex, male, *n* (%)	3,501 (57.7)	2,089 (57.4)	1,330 (54.8)
**Ethnicity**, ***n*** **(%)**			
White	4,182 (68.9)	2,505 (68.8)	1,730 (71.3)
Black	512 (8.4)	332 (9.1)	219 (9.0)
Hispanic	195 (3.2)	109 (3.0)	71 (2.9)
Asian	139 (2.3)	94 (2.6)	34 (1.4)
Other/Unknown	1,038 (17.1)	599 (16.5)	373 (15.4)
Baseline serum creatinine	1.1 (0.8–1.5)	1.0 (0.8–1.5)	1.1 (0.8–1.5)
Positive cultures^*^, ***n*** **(%)**			
Respiratory culture	960 (15.8)	603 (16.6)	347 (14.3)
Blood culture	648 (10.7)	391 (10.7)	229 (9.4)
Urine culture	1,044 (17.2)	616 (16.9)	375 (15.5)
Wound culture	213 (3.5)	134 (3.7)	75 (3.1)
Fluid culture	199 (3.3)	116 (3.2)	90 (3.7)
MRSA screen	310 (5.1)	189 (5.2)	109 (4.5)
Tissue	97 (1.6)	67 (1.8)	38 (1.6)
Anaerobic culture	108 (1.8)	64 (1.8)	41 (1.7)
Fungal culture	154 (2.5)	86 (2.4)	65 (2.7)
**KDIGO diagnostic criteria**, ***n*** **(%)**			
Serum creatinine	540 (8.9)	345 (9.5)	229 (9.4)
Urine output	3,467 (57.2)	2,035 (55.9)	1,368 (56.4)
Both	2,059 (33.9)	1,259 (34.6)	830 (34.2)
**Outcomes**			
In-hospital mortality^#^, *n* (%)	1,127 (18.6)	620 (17.0)	444 (18.3)
ICU length of stay	4 (3–8)	4 (3–8)	4 (3–8)
Hospital length of stay	10 (6–16)	9 (6–16)	9 (6–16)
Use of RRT^#^, *n* (%)	562 (9.3)	325 (8.9)	224 (9.2)

*MRSA, methicillin-resistant Staphylococcus aureus; KDIGO, kidney disease: improving global outcomes; ICU, intensive care unit; RRT, renal replacement therapy. Continuous variables were presented as median (interquartile range) and categorical variables were presented as n (%)*.

* *Positive cultures taken during the suspected infection time*.

# *In the first 28 days after ICU admission*.

### Model Performance

**Figure 2:**
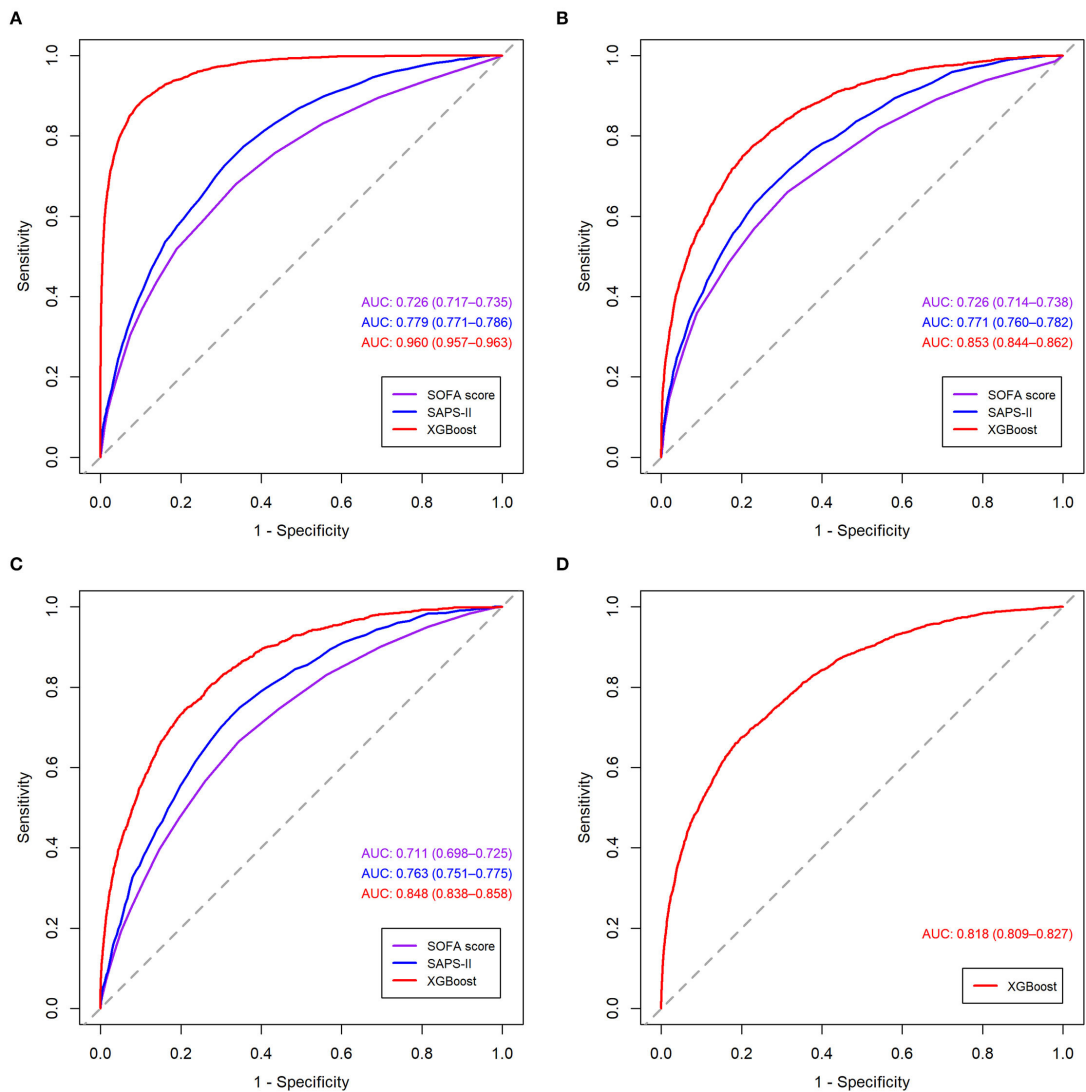
Receiver operating characteristic curves of the models for mortality in the following 48 h in the training set. **(A)** validation set; **(B)** internal test set; **(C)** external test set; **(D)** SOFA, sequential organ failure assessment; SAPS-II, simplified acute physiology score II; XGBoost, extreme gradient boosting.

**Figure 3:**
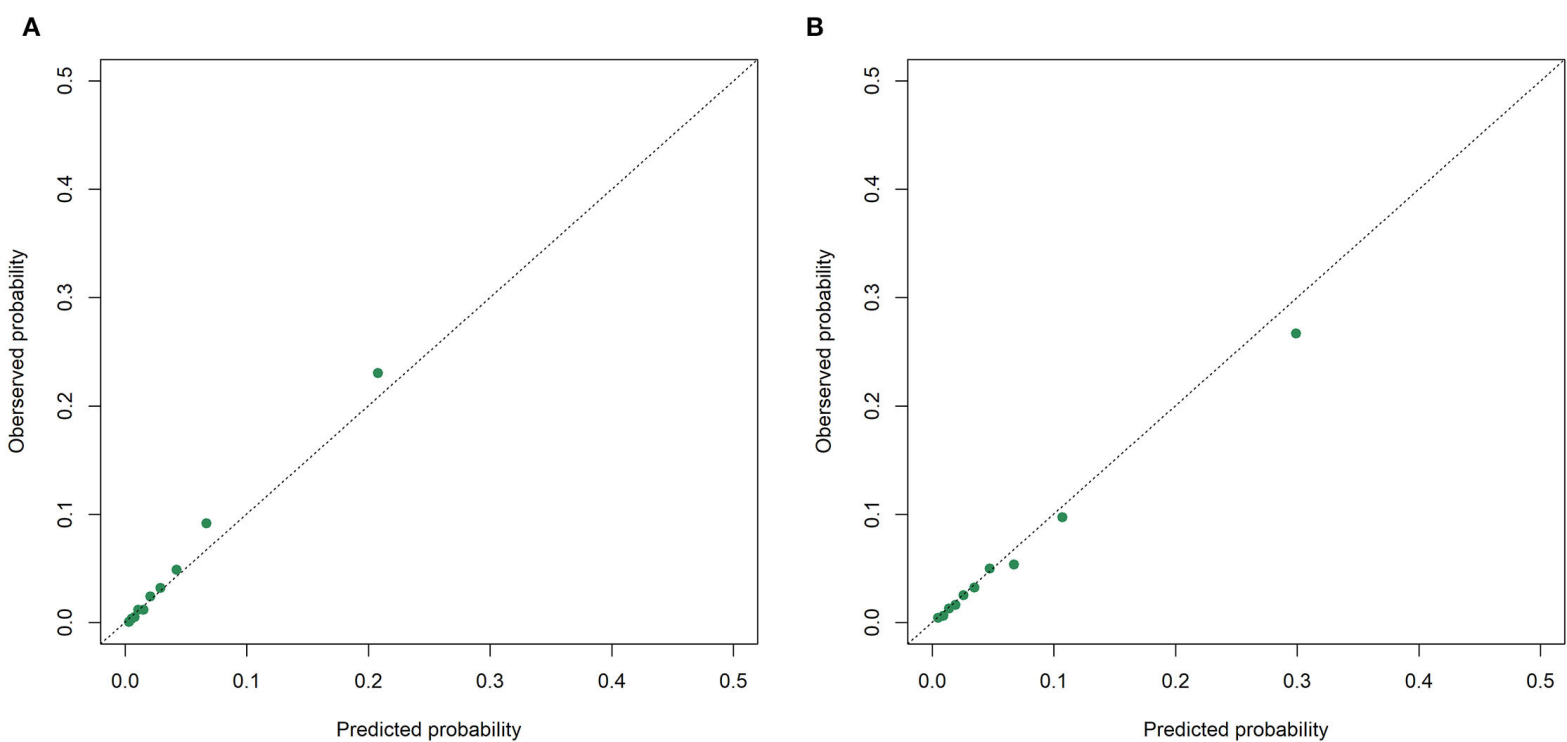
Calibration curves of the XGBoost model for mortality in the following 48 h in the internal. **(A)** and the external; **(B)** test sets.

The receiver operating characteristic curves of the models for mortality in the following 48, 72, and 120 h and in the first 28 days after ICU admission are shown in Figure 2 and Supplementary Figures S2–S4. The XGBoost models showed better discrimination than the SOFA score and SAPS-II, with the AUCs ranging from 0.848 to 0.804 in the internal test set and from 0.818 to 0.748 in the external test set. The sensitivity, specificity, and accuracy of the XGBoost models at different cutoffs for mortality prediction in the internal and the external test sets are provided in Table 3 and Supplementary Tables S6–S8. In the internal test set, the XGBoost model achieved a sensitivity of 80.1% and specificity of 72.9% at the cutoff of 0.0349 for mortality in the following 48 h. The sensitivity was slightly higher, and the specificity was lower in the external test set than in the internal test set across different cutoffs. The calibration curves of the XGBoost models comparing the predicted and observed probability across deciles in the internal and the external test sets are shown in Figure 3 and Supplementary Figures S5–S7. The XGBoost models were well-calibrated, except that they might underestimate or overestimate the probability at the higher risk deciles.

**Table 3:** Performance of the XGBoost model for mortality in the following 48 h at different cutoffs.

**Cutoffs**	**Sensitivity (%)**	**Specificity (%)**	**Accuracy (%)**
**Internal test set**			
0.0214	90.0	58.5	60.0
0.0280	85.0	66.6	67.4
0.0349	80.1	72.9	73.2
0.0431	75.0	78.3	78.1
0.0445^*^	74.3	79.1	78.9
0.0515	70.0	82.5	81.9
0.0600	65.1	85.6	84.7
0.0676	60.0	87.9	86.6
**External test set**			
0.0214	93.2	40.9	43.8
0.0280	89.2	50.5	52.7
0.0349	85.5	57.8	59.4
0.0431	81.1	64.7	65.7
0.0515	75.8	70.4	70.7
0.0600	71.6	75.0	74.8
0.0676	69.1	78.1	77.6
0.0735^*^	67.4	80.3	79.5

* *The cutoff value corresponding to the maximum Youden index (sensitivity + specificity - 1)*.

### Model Interpretability

**Figure 4:**
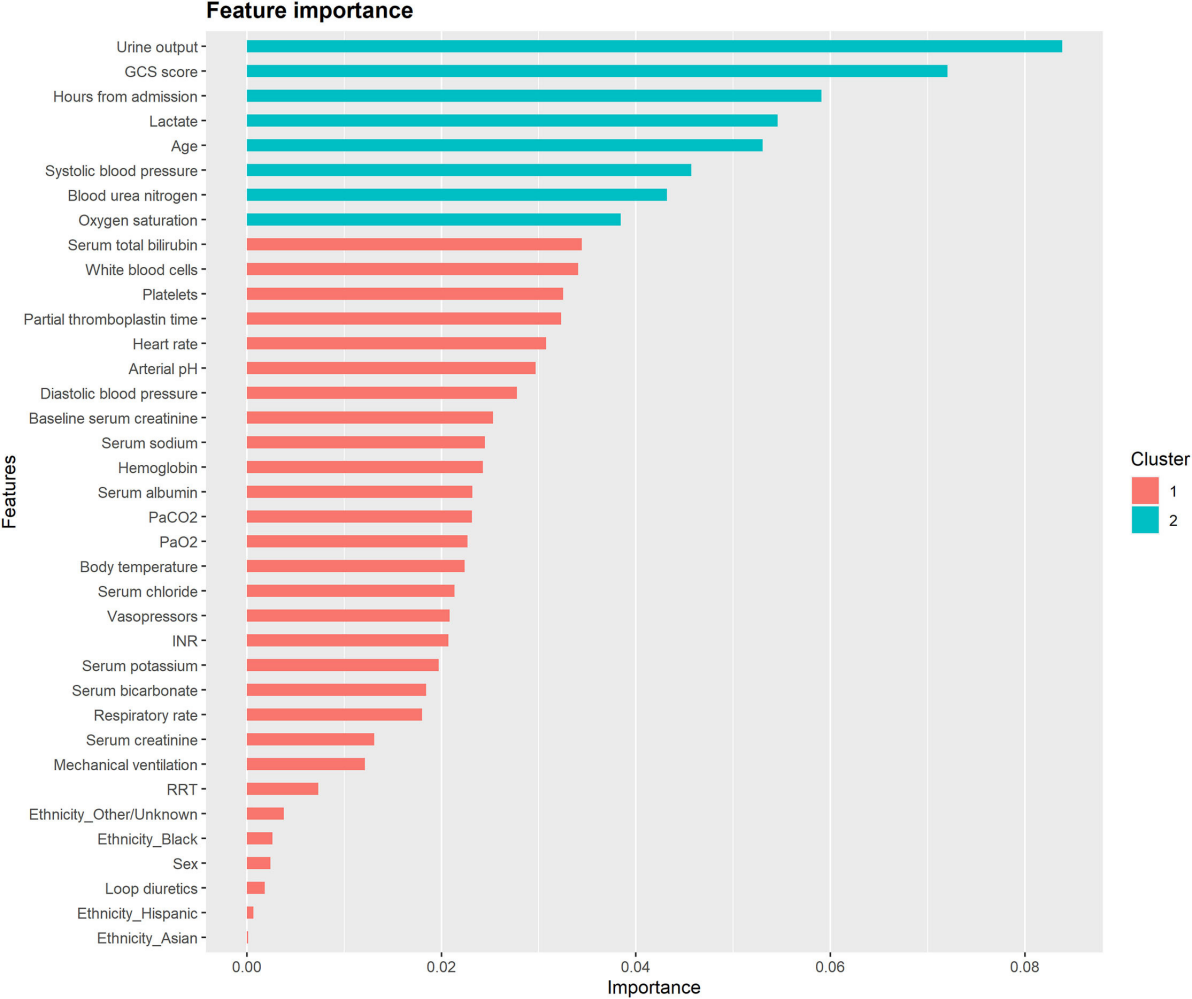
Feature importance derived from the XGBoost model for mortality in the following 48 h. The importance value represents the fractional contribution of each feature to the XGBoost model based on the total gain of this feature's splits. Higher percentage means a more important feature. GCS, glasgow coma scale; PaCO_2_, partial pressure of arterial carbon dioxide; PaO_2_, partial pressure of arterial oxygen; INR, international normalized ratio; RRT, renal replacement therapy.

**Figure 5:**
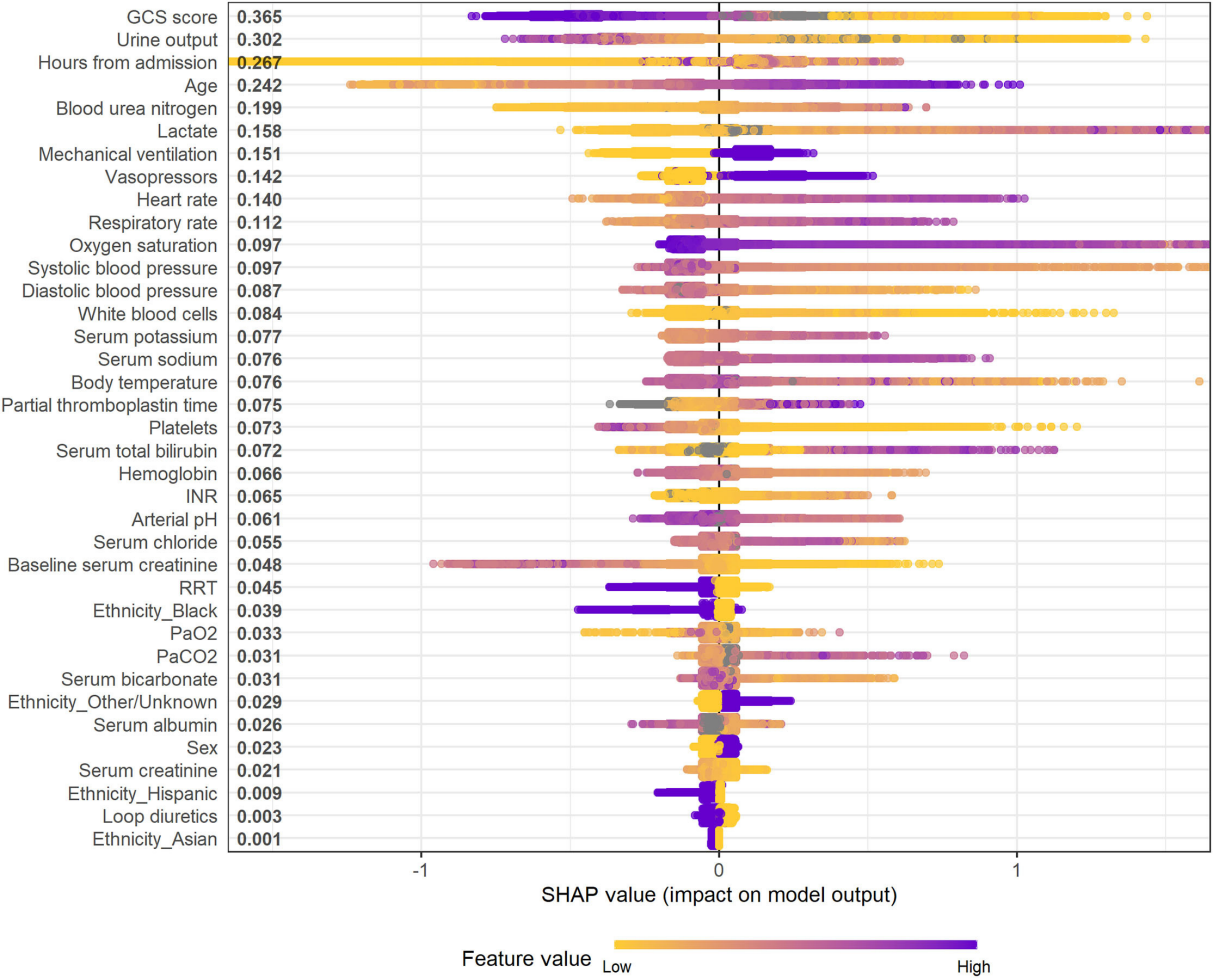
SHAP summary plot of the XGBoost model for mortality in the following 48 h. Higher SHAP value means a higher probability of death within the next 48 h. Purple represents higher feature values and yellow represents lower feature values. A dot is created for each feature attribution in calculating the output risk for each observation. GCS, glasgow coma scale; INR, international normalized ratio; RRT, renal replacement therapy; PaO_2_, partial pressure of arterial oxygen; PaCO_2_, partial pressure of arterial carbon dioxide.

Figure 4 and Supplementary Figures S8–S10 illustrate the feature importance derived from the XGBoost models. The top five most important predictor variables in the XGBoost model for mortality in the following 48 h were urine output, GCS score, hours from admission, serum lactate level, and age. Figure 5 and Supplementary Figures S11–S13 provide the SHAP summary plots of the XGBoost models, revealing the impact of the predictor variables on model output. Lower GCS score, decreased urine output, prolonged ICU length of stay, older age, and higher blood urea nitrogen (BUN) level were the top five factors associated with increased risk of death in the following 48 h.

### Sensitivity Analysis

In sensitivity analysis, the XGBoost models showed higher AUCs than the random forest and the support vector machine models in the internal and the external test sets (Supplementary Table S9). In addition, the XGBoost model using data gathered during the first 12 h after ICU admission showed poor predictive performance for in-hospital mortality in the first 28 days, with the AUC being 0.770 (95% CI 0.747**–**0.794) in the internal test set and 0.676 (95% CI 0.655**–**0.697) in the external test set (Supplementary Figure S14).

## Discussion

In this multi-center retrospective study, we developed and validated interpretable machine learning-based models using the XGBoost algorithm for real-time mortality prediction in critically ill patients with SA-AKI. The XGBoost models exhibited better performance than traditional risk scores (including the SOFA score and SAPS-II) or other machine learning models (including the random forest and support vector machine models) in predicting death in the following 48, 72, and 120 h and in the first 28 days after ICU admission. The XGBoost models could help identify high-risk patients in real time for early clinical interventions.

SA-AKI is common in critically ill patients with rapid clinical evolution and significantly higher mortality than those without AKI or with AKI attributed to other causes (6). Reliable prediction models are essential for clinicians to assess the risk of death and make proper clinical decisions in critically ill patients with SA-AKI. Generic scores, such as the SOFA score and SAPS-II, are widely used for outcome prediction in critical care settings. However, they have shown controversial results on predictive performance for mortality in AKI patients (7, 8, 30–32). Recently, several models have been proposed to predict AKI mortality in unselected ICU patients (31, 32), but few have been validated in patients with SA-AKI. Da Hora Passos et al. (7) proposed a clinical score to predict 7 days mortality in a cohort of 186 SA-AKI patients who required continuous RRT. The five-variable score showed better performance than the generic models, with a C-statistic of 0.82, but was limited to a single center and small sample size. In addition, Hu et al. (8) established a prediction model for in-hospital mortality in critically ill patients with SA-AKI. However, the model included only static clinical variables and showed insufficient predictive power.

Compared with the other risk prediction tools, our models have several strengths. First, the study demonstrated the applicability of the XGboost algorithm in mortality prediction in critically ill patients with SA-AKI. The XGBoost models had stronger predictive power than the traditional risk scores. Sensitivity analysis further showed that the XGBoost models were superior to the random forest and the support vector machine models. XGBoost-based models have shown exciting performance in various situations, such as volume responsiveness in patients with oliguric AKI (14), long-term kidney outcomes in patients with IgA nephrology (33), and mortality in ICU patients with rhabdomyolysis (34). The reasons for the improvement in predictive abilities observed in the XGBoost models may be multifactorial. The XGBoost algorithm, based on the gradient tree boosting framework, is adept at fitting non-linearities, discontinuities and complex high-order interactions. It is also robust to outliers in and multicollinearity among predictor variables. Besides, the XGBoost algorithm can handle missing values automatically, allowing the input of only available predictor variables in its clinical application.

Second, the real-time mortality prediction models can provide dynamic risk assessment and guide clinical decision-making. Patients in the ICU environment are clinically unstable, change rapidly between states of deterioration and improvement, and require continuous monitoring and interventions (35). It has promoted the establishment of real-time prediction models in critical care, such as models for mortality in critically ill children (35), the development of AKI (36), and sepsis onset (37, 38). Previously published models for mortality prediction in SA-AKI patients included static physiological parameters gathered during the early stages of the ICU stays. However, SA-AKI patients with similar disease severity at the early stage of ICU admission may exhibit different clinical outcomes due to distinct disease trajectories and treatment responses. The real-time prediction models can provide the risk of death updated on a 12-hour basis, which is more accurate and allows clinicians to make predictions dynamically.

Third, our models achieved promising predictive performance in both the internal and the external test sets, which demonstrated their robustness and generalizability. The predictor variables included in our model are routinely collected and usually available in the EHRs, and their values are rarely influenced by the examiner. Using only the most basic and commonly measured clinical data can facilitate the generalizability of the prediction model in other ICUs. Our models were further validated in an external test set, including 3,471 SA-AKI patients from a large multi-center critical care database with significantly different distributed features. Furthermore, automated data extraction from EHRs and data input can save additional labor and cost and reduce the possibility of incorrect entry in future clinical applications of the models (35).

Fourth, the interpretability of the models was explored to reveal the predictors for death over different time periods. Most recently, the relationship between the evolution of SA-AKI and mortality has been revealed. Uhel et al. (39) found that persistent AKI, but not transient AKI, was associated with increased mortality in critically ill septic patients. Ozrazgat-Baslanti et al. (40) also showed that persistent AKI and the absence of renal recovery were associated with worse clinical outcomes. Our results further demonstrated that decreased urine output and higher BUN level were important factors for increased real-time risk of death, suggesting the necessity for continuous renal function monitoring in SA-AKI patients. Additionally, the discovery of other potentially modifiable extra-renal risk factors, such as lower GCS score, higher lactate level, higher heart rate, and higher respiratory rate, may help improve patient care and outcomes.

Our study was subject to some limitations. Firstly, it was a retrospective analysis based on the publicly accessible databases. The diagnosis of sepsis in the eICU-CRD may not meet the updated Sepsis-3 criteria. It remains unclear whether the prediction model performs well for individual prognostication and whether its clinical application can improve patient outcomes. Secondly, although the XGBoost algorithm can handle missing values automatically, the presence of missing data may lead to bias. Thirdly, clinical data beyond the ICU stays were unavailable, limiting the continuous assessment of the risk of death for SA-AKI patients who were transferred to the general wards or other locations. Finally, the visualization and application of the models are still limited. In our subsequent study, we will prospectively investigate the effectiveness of our models and develop a web-based risk calculator that automatically extracts data from EHRs and performs risk calculations.

## Conclusions

This study developed and externally validated interpretable machine learning XGBoost models for real-time mortality prediction in critically ill patients with SA-AKI. The XGBoost models, based on routine clinical variables updated every 12 h, showed promising performance in predicting death in the following 48, 72, and 120 h and in the first 28 days after ICU admission. The real-time prediction models are useful tools for early identification of high-risk patients and timely clinical interventions. Future studies are required to determine the robustness and effectiveness of the prediction models in a prospective way.

## Data Availability Statement

The datasets analyzed for this study can be found in the MIMIC-IV (https://mimic.mit.edu/) and eICU-CRD (https://eicu-crd.mit.edu/).

## Ethics Statement

The studies involving human participants were reviewed and approved by the Institutional Review Boards of the Beth Israel Deaconess Medical Center and Massachusetts Institute of Technology. Written informed consent for participation was not required for this study in accordance with the National Legislation and the Institutional Requirements.

## Author Contributions

S-BD designed, supervised the study, and drafted the manuscript. X-QL performed the data extraction, analysed, interpreted the data, and drafted the manuscript. PY and Y-XK analyzed and interpreted the data and critically revised the manuscript. Y-HD, TW, and XW analyzed the data and revised the manuscript critically for important intellectual content. All authors have read and approved the final manuscript.

## Funding

This study was supported by National Natural Science Foundation of China (Grant No. 81873607).

## Conflict of Interest

The authors declare that the research was conducted in the absence of any commercial or financial relationships that could be construed as a potential conflict of interest.

## Publisher's Note

All claims expressed in this article are solely those of the authors and do not necessarily represent those of their affiliated organizations, or those of the publisher, the editors and the reviewers. Any product that may be evaluated in this article, or claim that may be made by its manufacturer, is not guaranteed or endorsed by the publisher.
