# Supplementary material for: Development and Validation of Machine Learning Models for Real-Time Mortality Prediction in Critically Ill Patients With Sepsis-Associated Acute Kidney Injury
Source: Front Med (Lausanne). 2022 Jun 15;9:853102. doi: 10.3389/fmed.2022.853102 (PMC9240603; doi:10.3389/fmed.2022.853102)
Supplement: Supplementary file 1 [file Data_Sheet_1.PDF]

Table S1. Percentages of missing values in the predictor variables.

Features	Training dataset (%)	Validation set (%)	Internal test set (%)	External test set (%)
Age	0.00	0.00	0.00	0.00
Sex	0.00	0.00	0.00	0.01
Ethnicity	0.00	0.00	0.00	0.00
Hours from admission	0.00	0.00	0.00	0.00
Systolic blood pressure	0.12	0.07	0.13	5.30
Diastolic blood pressure	0.12	0.07	0.13	5.28
Heart rate	0.12	0.04	0.13	4.54
Respiratory rate	0.11	0.05	0.13	5.60
Body temperature	2.53	2.52	2.51	2.13
Oxygen saturation	0.12	0.06	0.13	18.93
GCS score	14.77	15.26	14.56	16.71
Urine output	6.22	6.07	6.62	35.34
Baseline serum creatinine	0.03	0.28	0.00	0.09
Hemoglobin	0.68	0.73	0.69	3.16
White blood cells	0.70	0.76	0.72	3.55
Platelets	0.69	0.76	0.72	3.75
Serum total bilirubin	25.56	26.27	24.23	20.82
Serum albumin	40.02	41.81	37.93	16.45
Serum creatinine	0.49	0.52	0.45	2.38
Blood urea nitrogen	0.49	0.52	0.46	2.38
Arterial pH	15.55	15.80	14.57	26.94
PaO_2	15.67	16.00	14.66	25.75
$PaCO_2$	15.67	16.00	14.66	26.58
Serum sodium	0.44	0.48	0.38	2.25
Serum potassium	0.41	0.45	0.36	2.15
Serum chloride	0.44	0.48	0.38	2.40
Serum bicarbonate	0.48	0.53	0.46	6.66
Lactate	9.62	9.83	9.15	24.03
INR	4.15	4.27	4.15	34.54
Partial thromboplastin time	4.34	4.49	4.49	52.28
Mechanical ventilation	0.00	0.00	0.00	0.00
Vasopressors	0.00	0.00	0.00	0.00
Renal replacement therapy	0.00	0.00	0.00	0.00
Loop diuretics	0.00	0.00	0.00	0.00

GCS, Glasgow Coma Scale; PaO_2, partial pressure of arterial oxygen; $PaCO_2$, partial pressure of arterial carbon dioxide; INR, international normalized ratio.

Table S2. Functions and parameters used for the XGBoost algorithm.

Models	Function	Parameters
Model for mortality in the following 48 hours	xgb.train	booster = "gbtree", eta = 0.01, min_child_weight = 3, max_depth = 6, gamma = 0.4, subsample = 0.8, colsample_bytree = 0.8, max_delta_step = 1, objective = "binary:logistic", nrounds = 1035
Model for mortality in the following 72 hours	xgb.train	booster = "gbtree", eta = 0.01, min_child_weight = 3, max_depth = 7, gamma = 0.3, subsample = 0.8, colsample_bytree = 0.8, max_delta_step = 1, objective = "binary:logistic", nrounds = 866
Model for mortality in the following 120 hours	xgb.train	booster = "gbtree", eta = 0.01, min_child_weight = 3, max_depth = 3, gamma = 0, subsample = 0.8, colsample_bytree = 0.8, max_delta_step = 1, objective = "binary:logistic", nrounds = 3045
Model for mortality in the first 28 days after ICU admission	xgb.train	booster = "gbtree", eta = 0.01, min_child_weight = 5, max_depth = 9, gamma = 0.1, subsample = 0.8, colsample_bytree = 0.8, max_delta_step = 1, objective = "binary:logistic", nrounds = 775

Table S3. Baseline characteristics and outcomes of SA-AKI patients in the external test set.

Variables	External test set (n = 3471)
Age (year)	68 (58-78)
Sex, male, n (%)	1838 (53.0)
Ethnicity, n (%)	
White	2694 (77.6)
Black	389 (11.2)
Hispanic	156 (4.5)
Asian	39 (1.1)
Other/Unknown	193 (5.6)
Baseline serum creatinine	1.5 (0.9-2.4)
Infection site, n (%)	
Pulmonary	1360 (39.2)
Renal/urinary tract infection	692 (19.9)
Gastrointestinal	500 (14.4)
Cutaneous/soft tissue	290 (8.4)
Gynecologic	7 (0.2)
Other	241 (6.9)
Unknown	381 (11.0)
KDIGO diagnostic criteria, n (%)	
Serum creatinine	1421 (40.9)
Urine output	1337 (38.5)
Both	713 (20.5)
Outcomes	
In-hospital mortality[#], n (%)	789 (22.7)
ICU length of stay	4 (3-8)
Hospital length of stay	9 (6-15)
Use of RRT[#], n (%)	566 (16.3)

KDIGO, Kidney Disease: Improving Global Outcomes; ICU, intensive care unit; RRT, renal replacement therapy.

[#]In the first 28 days after ICU admission.

Table S4. Outcomes in each 12-hour window of the ICU stays.

Number	Training set		Validation set		Internal test set		External test set	
	Patients present in the ICU	In-hospital mortality in the first 28 days	Patients present in the ICU	In-hospital mortality in the first 28 days	Patients present in the ICU	In-hospital mortality in the first 28 days	Patients present in the ICU	In-hospital mortality in the first 28 days
1	6066	1127	3639	620	2427	444	3471	789
2	6066	1127	3639	620	2427	444	3471	789
3	6066	1127	3639	620	2427	444	3471	789
4	6066	1127	3639	620	2427	444	3471	789
5	5070	1015	3071	555	2016	402	3003	683
6	4402	913	2683	498	1760	366	2519	601
7	3814	827	2282	448	1511	336	2191	532
8	3356	730	2028	400	1338	305	1897	469
9	2938	656	1757	353	1174	272	1690	423
10	2645	598	1572	327	1051	242	1490	369
11	2399	544	1435	308	930	219	1326	331
12	2168	497	1304	278	850	204	1176	292
13	1963	450	1194	253	779	190	1058	265
14	1795	414	1082	227	722	175	947	243
15	1633	369	985	209	664	161	880	222
16	1519	344	902	190	610	151	787	201
17	1401	321	824	173	572	142	729	189
18	1289	294	754	160	517	130	656	170
19	1204	278	695	149	480	122	617	159
20	1102	250	644	136	436	109	550	141

21	1037	237	605	129	407	101	506	127
22	965	215	551	116	385	94	442	110
23	903	197	512	110	365	86	409	101
24	834	174	492	104	338	80	366	86
25	778	157	458	95	316	77	344	79
26	733	144	418	83	289	66	319	74
27	679	132	386	75	263	56	293	66
28	622	115	363	70	244	51	267	58
29	581	107	338	59	226	46	244	52
30	540	95	316	54	211	43	222	43
31	506	85	290	51	197	41	208	40
32	462	79	276	46	182	34	197	37
33	440	72	264	46	171	30	183	33
34	414	64	245	41	157	26	163	29
35	396	60	237	40	149	26	150	27
36	371	49	221	35	139	22	138	26
37	347	44	210	32	134	20	131	23
38	319	38	193	28	127	19	123	18
39	309	37	182	27	118	16	116	17
40	291	33	165	23	109	16	105	17
41	276	29	154	20	105	15	96	14
42	259	28	145	17	95	13	84	11
43	246	23	132	14	91	12	72	6
44	229	20	122	12	88	10	68	5
45	213	16	117	11	84	9	63	4

46	198	15	109	10	78	9	61	4
47	190	13	103	8	73	6	57	3
48	181	11	98	5	70	6	56	3
49	176	10	95	5	62	6	56	3
50	169	10	86	4	59	4	52	2
51	157	8	81	4	54	4	49	2
52	144	7	79	3	49	4	46	2
53	135	6	73	2	44	1	37	1
54	126	2	69	0	42	1	34	0
55	122	1	66	0	41	1	33	0
56	117	0	62	0	38	0	30	0
Total	77427	15341	46081	8523	30718	6353	41220	9569

ICU, intensive care unit.

Table S5. Distribution of the predictor variables within each 12-hour window of the ICU stays.

Variables	Training set (n = 77427)	Validation set (n = 46081)	Internal test set (n = 30718)	External test set (n = 41220)
Age (year)	68 (56-78)	68 (58-78)	68 (58-78)	67 (56-77)
Sex, n (%)	45511 (58.8)	27218 (59.1)	17393 (56.6)	22024 (53.4)
Ethnicity, n (%)				
White	51756 (66.8)	30969 (67.2)	21438 (69.8)	31260 (75.8)
Black	6494 (8.4)	4110 (8.9)	2878 (9.4)	4922 (11.9)
Hispanic	2706 (3.5)	1444 (3.1)	782 (2.5)	1876 (4.6)
Asian	1780 (2.3)	1164 (2.5)	409 (1.3)	575 (1.4)
Other/Unknown	14691 (19.0)	8394 (18.2)	5211 (17.0)	2587 (6.3)
Hours from admission (hour)	96 (48-204)	96 (48-192)	96 (48-192)	84 (36-168)
Systolic blood pressure (mmHg)	117 (106-130)	116 (106-130)	116 (106-130)	116 (105-132)
Diastolic blood pressure (mmHg)	60 (53-68)	60 (53-68)	60 (53-68)	61 (55-70)
Heart rate (bpm)	87 (76-99)	86 (75-98)	86 (75-98)	91 (79-103)
Respiratory rate (bpm)	20 (17-24)	20 (17-24)	20 (17-24)	21 (18-25)
Body temperature (°C)	36.9 (36.6-37.3)	36.9 (36.6-37.3)	36.9 (36.6-37.3)	36.9 (36.5-37.3)
Oxygen saturation (%)	97 (95-99)	97 (95-98)	97 (95-98)	97 (95-99)
GCS score	14 (10-15)	14 (10-15)	14 (10-15)	11 (9-15)
Urine output (mL)	750 (410-1260)	760 (425-1270)	740 (418-1238)	545 (225-1015)
Baseline serum creatinine (mg/dL)	1.1 (0.8-1.6)	1.1 (0.8-1.5)	1.1 (0.8-1.6)	1.5 (0.9-2.5)
Hemoglobin (g/dL)	9.4 (8.3-10.6)	9.3 (8.3-10.6)	9.4 (8.3-10.6)	9.3 (8.3-10.7)
White blood cells ($\times 10^9$/L)	11.3 (8.2-15.4)	11.3 (8.3-15.3)	11.3 (8.2-15.5)	12.7 (8.7-18.3)
Platelets ($\times 10^9$/L)	178 (112-270)	183 (116-275)	174 (110-261)	158 (91-239)
Serum total bilirubin (mg/dL)	0.8 (0.4-1.9)	0.7 (0.4-1.7)	0.8 (0.4-2.1)	0.8 (0.5-1.7)
Serum albumin (g/dL)	2.8 (2.4-3.2)	2.8 (2.4-3.2)	2.8 (2.3-3.2)	2.3 (1.9-2.7)
Serum creatinine (mg/dL)	1.1 (0.7-1.9)	1.1 (0.8-1.8)	1.2 (0.8-1.9)	1.6 (0.9-2.8)
Blood urea nitrogen (mg/dL)	28 (17-46)	27 (17-46)	29 (18-47)	37 (23-56)
Arterial pH	7.41 (7.36-7.45)	7.41 (7.36-7.46)	7.41 (7.36-7.45)	7.39 (7.33-7.44)
PaO_2 (mmHg)	104 (84-133)	103 (84-133)	104 (85-132)	89 (73-115)
$PaCO_2$ (mmHg)	40 (35-46)	40 (35-46)	40 (35-45)	38 (33-45)
Serum sodium (mmol/L)	139 (136-143)	139 (136-143)	139 (136-143)	140 (136-144)
Serum potassium (mmol/L)	4.0 (3.7-4.4)	4.0 (3.7-4.4)	4.0 (3.7-4.4)	3.9 (3.6-4.4)
Serum chloride (mmol/L)	104 (100-108)	104 (100-108)	104 (100-109)	106 (101-111)
Serum bicarbonate (mmol/L)	24 (21-28)	25 (21-28)	24 (21-28)	24 (21-27)
Lactate (mmol/L)	1.4 (1.1-1.9)	1.4 (1.0-1.9)	1.4 (1.1-1.9)	1.7 (1.2-2.5)
INR	1.3 (1.2-1.6)	1.3 (1.1-1.5)	1.3 (1.2-1.6)	1.4 (1.2-1.7)
Partial thromboplastin time (sec)	32.5 (28.2-43.0)	32.0 (28.0-41.5)	32.6 (28.3-43.0)	36.0 (30.8-45.0)
Mechanical ventilation, n (%)	45758 (59.1)	27132 (58.9)	18250 (59.4)	22487 (54.6)
Vasopressors, n (%)	22573 (29.2)	13907 (30.2)	9400 (30.6)	17554 (42.6)

Renal replacement therapy, n (%)	8697 (11.2)	4561 (9.9)	3312 (10.8)	2908 (7.1)
Loop diuretics, n (%)	32012 (41.3)	19236 (41.7)	12844 (41.8)	8335 (20.2)

GCS, Glasgow Coma Scale; PaO_2, partial pressure of arterial oxygen; $PaCO_2$, partial pressure of arterial carbon dioxide; INR, international normalized ratio; RRT, renal replacement therapy

Table S6. Performance of the XGBoost model for mortality in the following 72 hours at different cutoffs.

Cutoffs	Sensitivity (%)	Specificity (%)	Accuracy (%)
Internal test set			
0.0279	90.0	51.7	54.3
0.0393	85.0	62.9	64.4
0.0481	80.0	69.5	70.2
0.0566	75.0	74.4	74.4
0.0663	70.0	78.5	78.0
0.0735*	67.4	80.3	79.5
0.0760	65.0	82.0	80.9
0.0872	60.0	85.1	83.4
External test set			
0.0279	93.9	34.1	39.1
0.0393	88.8	47.1	50.6
0.0481	84.7	54.8	57.3
0.0566	80.6	60.6	62.3
0.0663	76.4	66.3	67.1
0.0760	72.3	71.0	71.1
0.0872	68.4	75.2	74.6
0.0879*	68.2	75.4	74.8

*The cutoff value corresponding to the maximum Youden index (sensitivity + specificity - 1).

Table S7. Performance of the XGBoost model for mortality in the following 120 hours at different cutoffs.

Cutoffs	Sensitivity (%)	Specificity (%)	Accuracy (%)
Internal test set			
0.0468	90.0	50.4	54.5
0.0594	85.0	58.4	61.2
0.0739	80.0	65.6	67.1
0.0848*	76.8	69.9	70.7
0.0889	75.0	71.4	71.8
0.1029	70.0	75.6	75.0
0.1164	65.0	78.9	77.5
0.1321	60.0	82.1	79.8
External test set			
0.0468	92.0	34.5	41.7
0.0594	88.5	42.9	48.6
0.0739	84.6	50.8	55.0
0.0889	79.9	57.9	60.6
0.1029	76.1	63.0	64.6
0.1164	72.5	67.1	67.8
0.1315*	68.8	71.3	71.0
0.1321	68.6	71.4	71.1

*The cutoff value corresponding to the maximum Youden index (sensitivity + specificity - 1).

Table S8. Performance of the XGBoost model for mortality in the first 28 days at different cutoffs.

Cutoffs	Sensitivity (%)	Specificity (%)	Accuracy (%)
Internal test set			
0.0847	90.0	51.1	59.1
0.1101	85.0	59.4	64.7
0.1363	80.0	65.5	68.5
0.1633	75.0	70.7	71.6
0.1756*	73.2	72.7	72.8
0.1923	70.0	75.2	74.1
0.2197	65.0	79.0	76.1
0.2451	60.0	81.9	77.4
External test set			
0.0847	92.7	30.6	45.0
0.1101	89.4	38.8	50.5
0.1363	85.0	46.5	55.5
0.1633	80.7	53.5	59.8
0.1923	75.1	59.8	63.3
0.2197	70.6	64.7	66.1
0.2451	66.5	69.0	68.4
0.2505*	65.8	69.8	68.9

*The cutoff value corresponding to the maximum Youden index (sensitivity + specificity - 1).

Table S9. Performance of the machine learning models for mortality prediction

Models	Internal test set		External test set	
	AUC	95% CI	AUC	95% CI
Mortality in the following 48 hours				
XGBoost	0.848	0.838-0.858	0.818	0.809-0.827
Random forest*	0.829	0.819-0.840	0.786	0.776-0.796
SVM*	0.754	0.740-0.769	0.730	0.719-0.742
Mortality in the following 72 hours				
XGBoost	0.827	0.818-0.835	0.795	0.787-0.803
Random forest*	0.812	0.802-0.821	0.769	0.760-0.777
SVM*	0.739	0.726-0.751	0.721	0.711-0.730
Mortality in the following 120 hours				
XGBoost	0.808	0.801-0.816	0.768	0.761-0.775
Random forest*	0.797	0.789-0.805	0.752	0.745-0.759
SVM*	0.740	0.730-0.750	0.723	0.715-0.731
Mortality in the first 28 days				
XGBoost	0.804	0.798-0.810	0.748	0.742-0.753
Random forest*	0.798	0.792-0.804	0.745	0.740-0.751
SVM*	0.767	0.760-0.773	0.720	0.715-0.726

AUC, area under the receiver operating characteristic curve; CI, confidence interval; XGBoost, eXtreme Gradient Boosting; SVM, support vector machine.

*The median (for continuous variables) or mode (for categorical variables) by location was imputed for missing values before model development and validation.

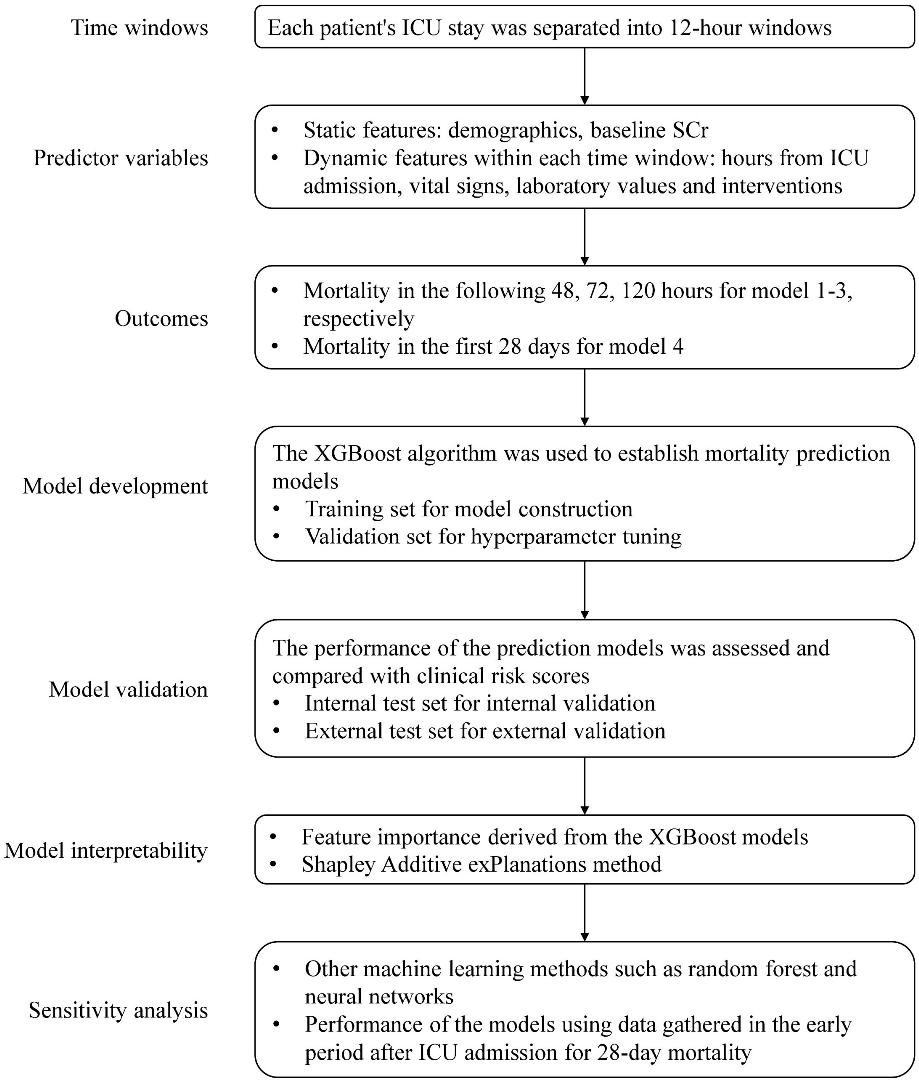

Figure S1. Diagram of methods. ICU, intensive care unit; SCr, Serum creatinine; XGBoost, eXtreme Gradient Boosting.

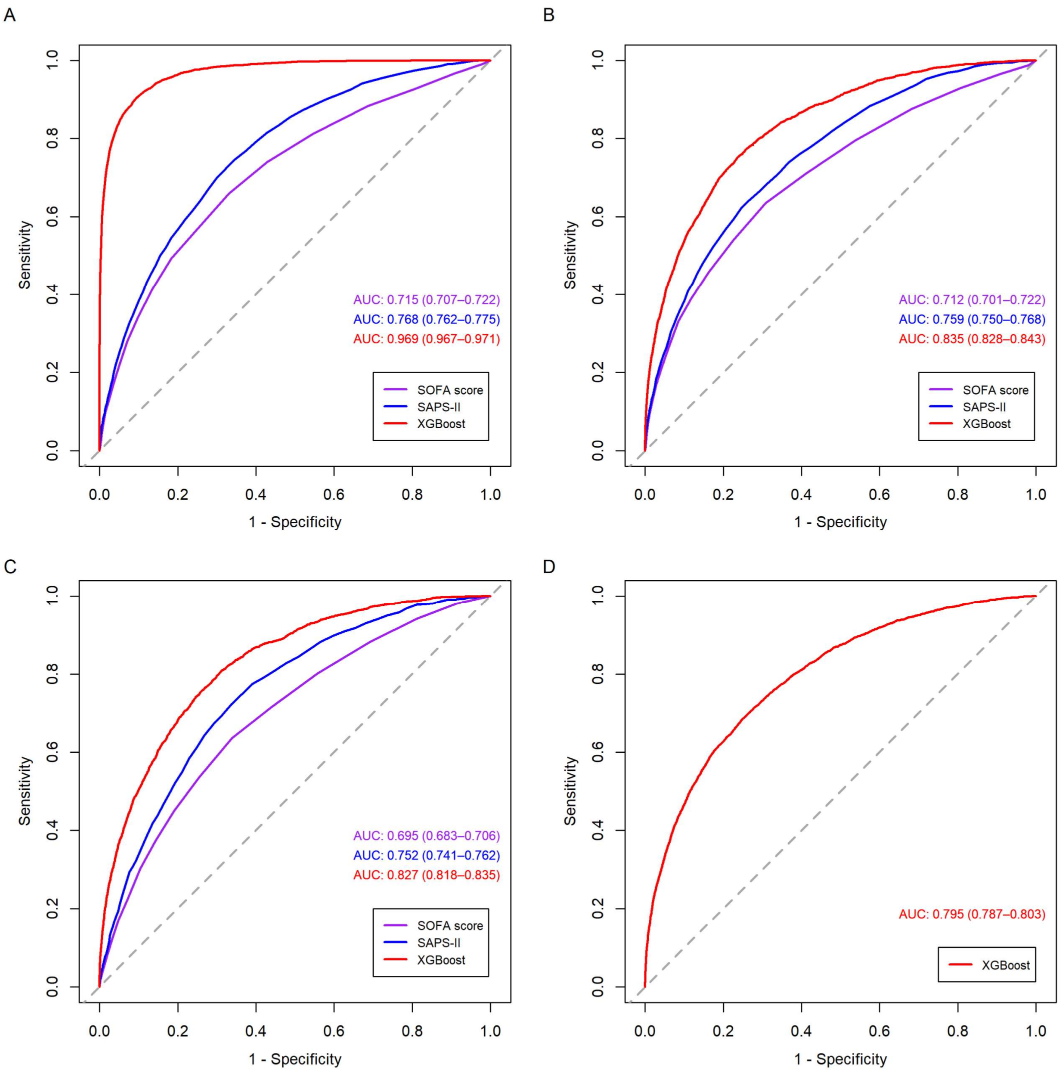

Figure S2. Receiver operating characteristic curves of the models for mortality in the following 72 hours in the training set (A), validation set (B), internal test set (C) and external test set (D). SOFA, Sequential Organ Failure Assessment; SAPS-II, Simplified Acute Physiology Score II; XGBoost, eXtreme Gradient Boosting.

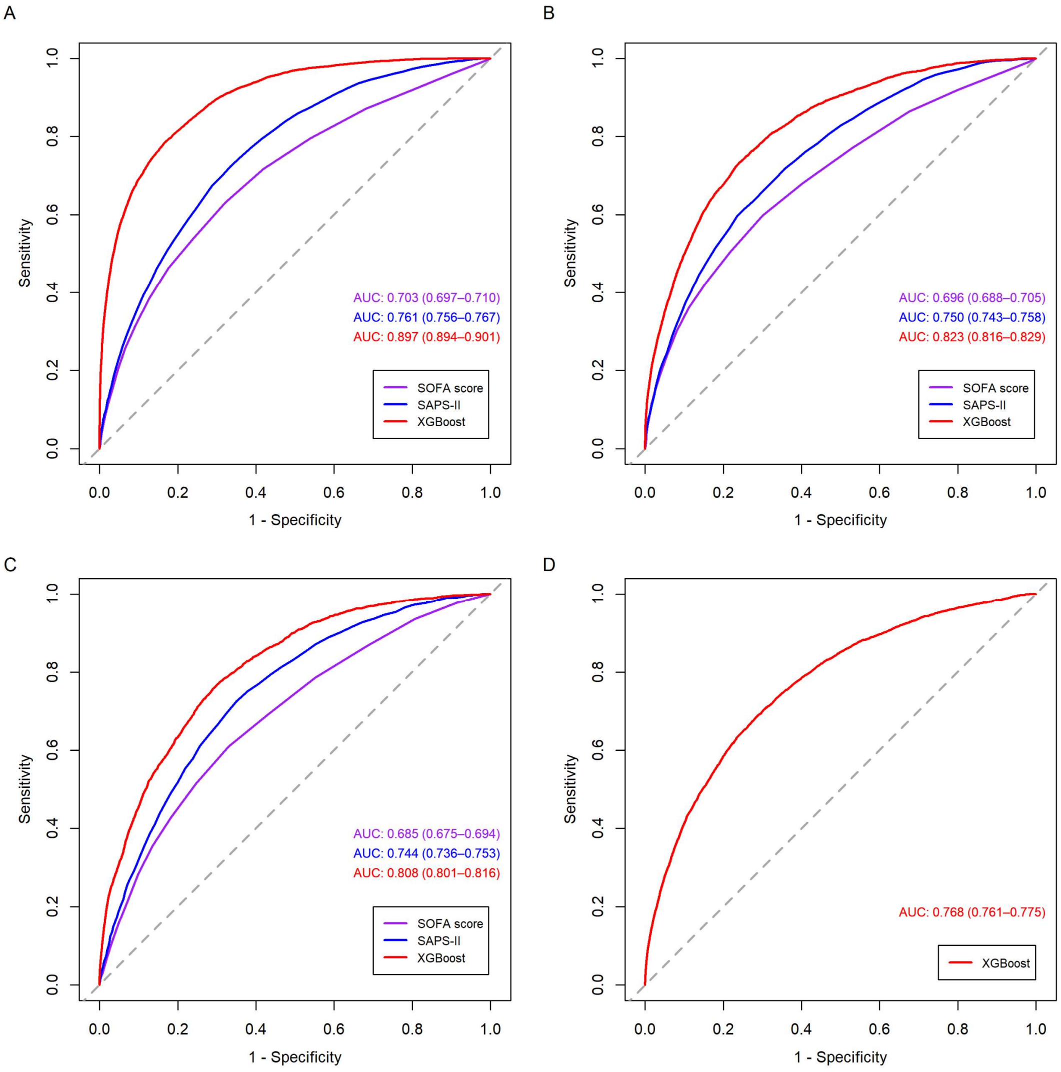

Figure S3. Receiver operating characteristic curves of the models for mortality in the following 120 hours in the training set (A), validation set (B), internal test set (C) and external test set (D). SOFA, Sequential Organ Failure Assessment; SAPS-II, Simplified Acute Physiology Score II; XGBoost, eXtreme Gradient Boosting.

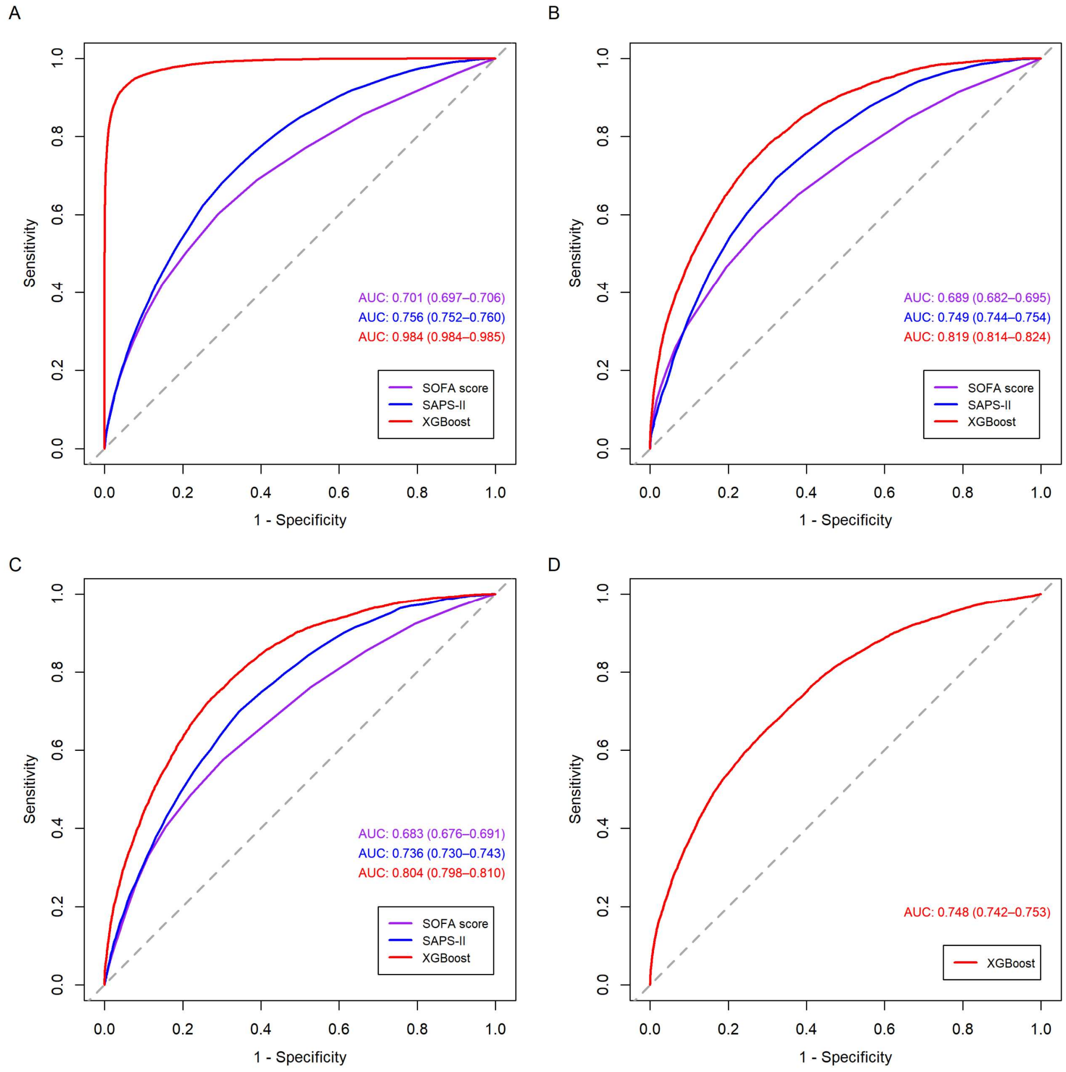

Figure S4. Receiver operating characteristic curves of the models for mortality in the first 28 days in the training set (A), validation set (B), internal test set (C) and external test set (D). SOFA, Sequential Organ Failure Assessment; SAPS-II, Simplified Acute Physiology Score II; XGBoost, eXtreme Gradient Boosting.

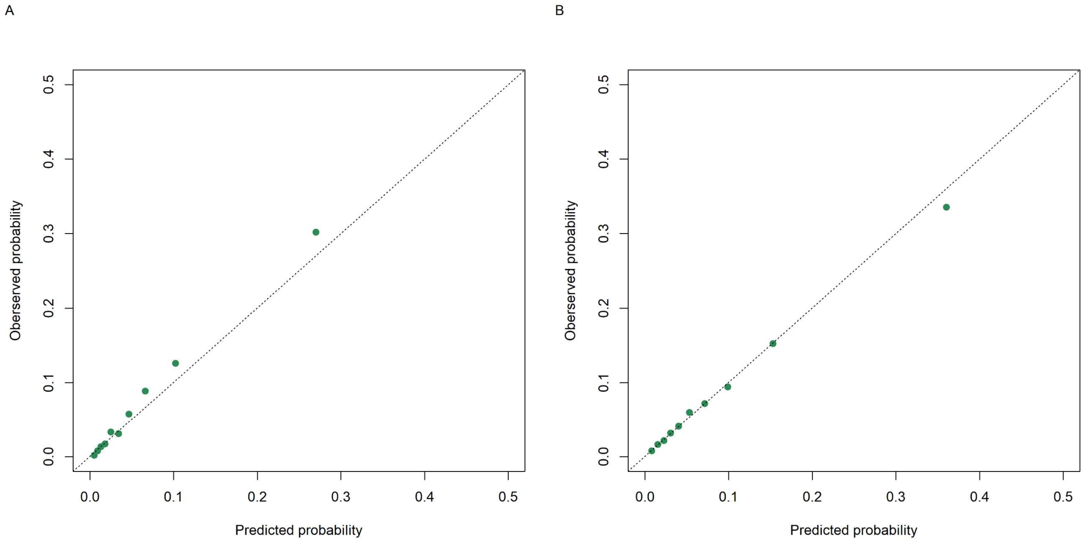

Figure S5. Calibration curves of the XGBoost model for mortality in the following 72 hours in the internal (A) and the external (B) test sets.

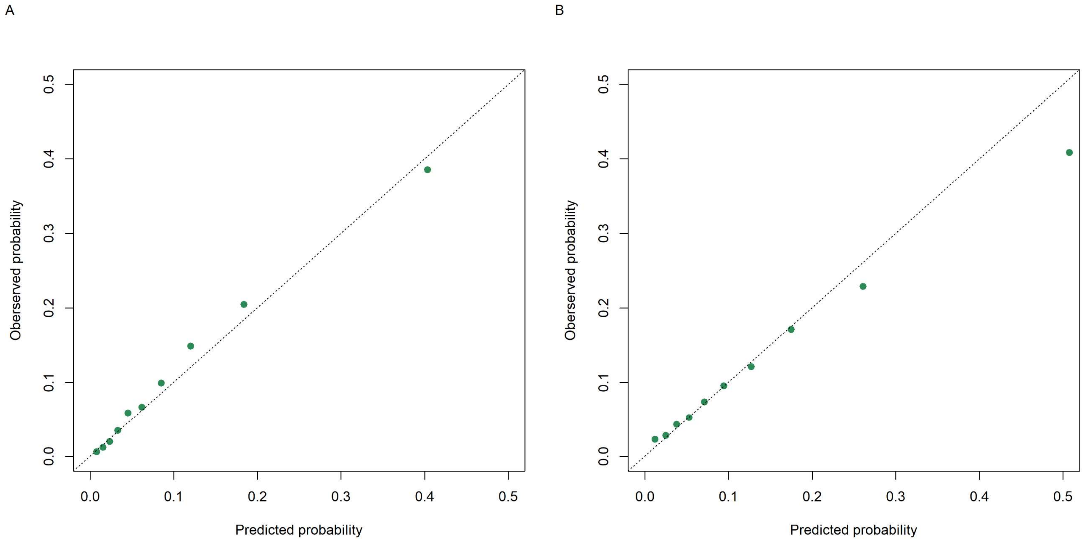

Figure S6. Calibration curves of the XGBoost model for mortality in the following 120 hours in the internal (A) and the external (B) test sets.

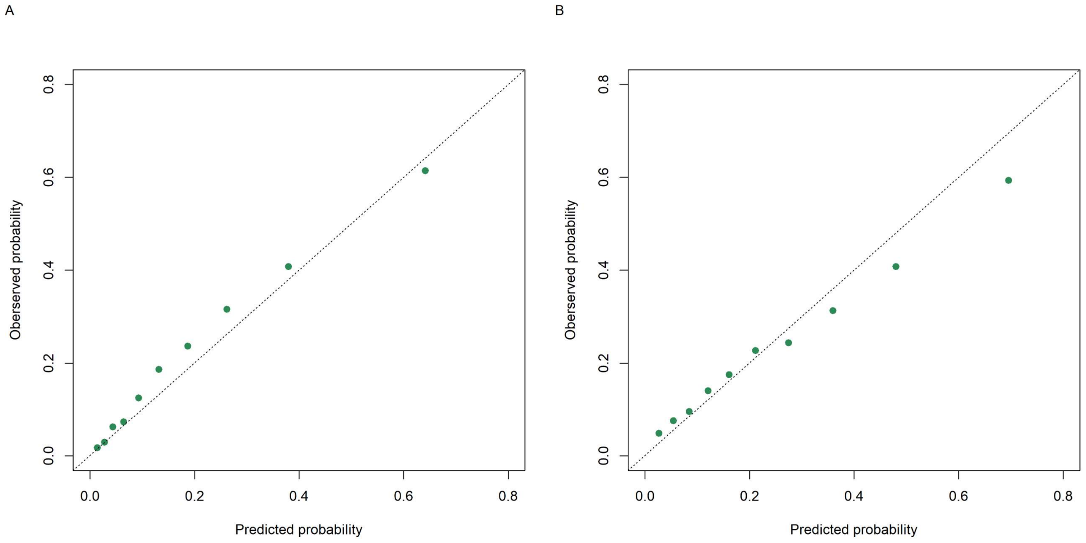

Figure S7. Calibration curves of the XGBoost model for mortality in the first 28 days in the internal (A) and the external (B) test sets.

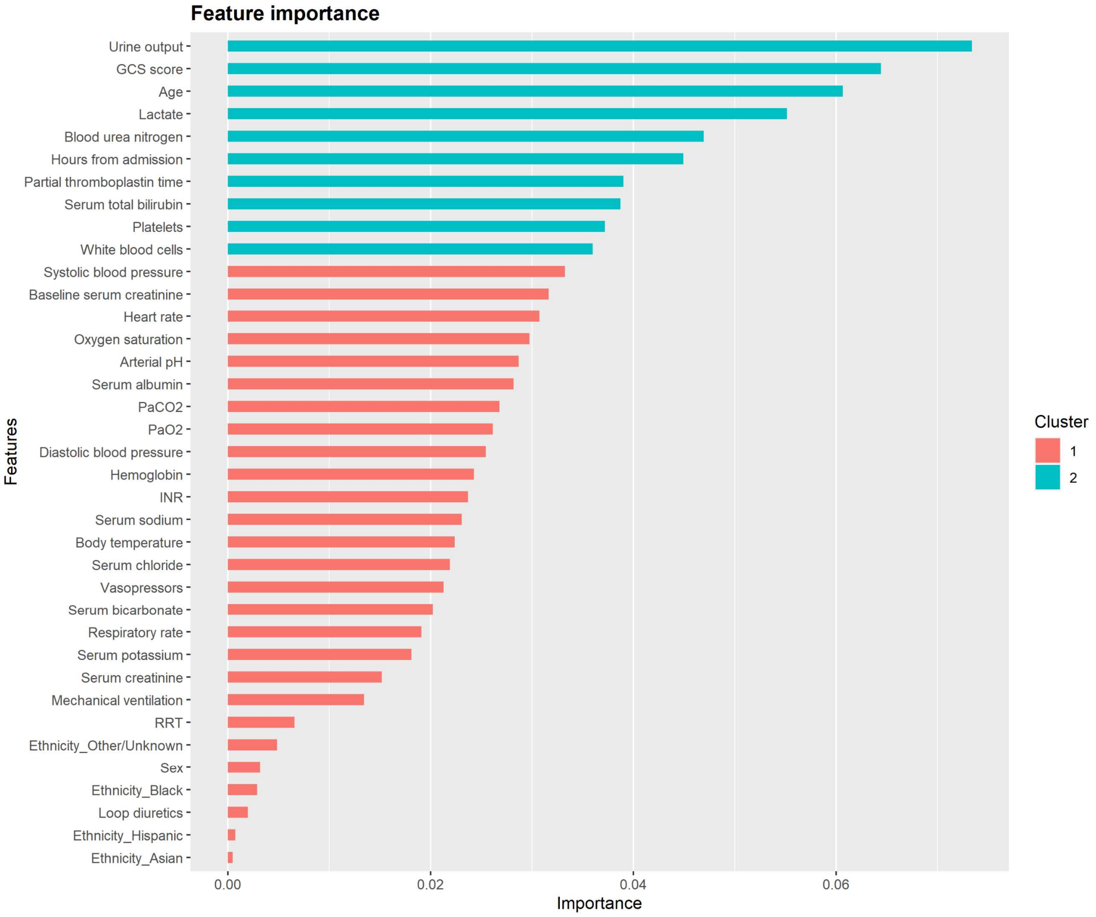

Figure S8. Feature importance derived from the XGBoost model for mortality in the following 72 hours. GCS, Glasgow Coma Scale; PaCO2, partial pressure of arterial carbon dioxide; PaO2, partial pressure of arterial oxygen; INR, international normalized ratio; RRT, renal replacement therapy.

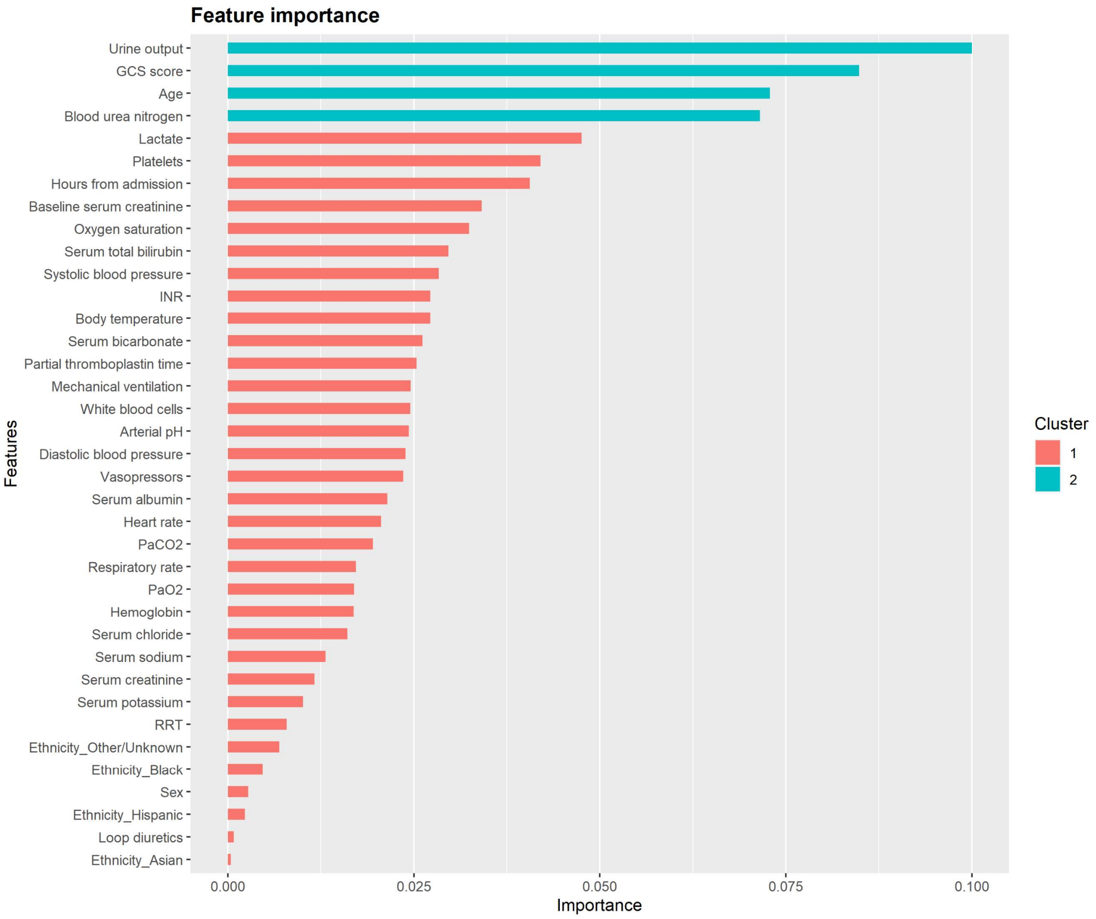

Figure S9. Feature importance derived from the XGBoost model for mortality in the following 120 hours. GCS, Glasgow Coma Scale; INR, international normalized ratio; PaCO2, partial pressure of arterial carbon dioxide; PaO2, partial pressure of arterial oxygen; RRT, renal replacement therapy.

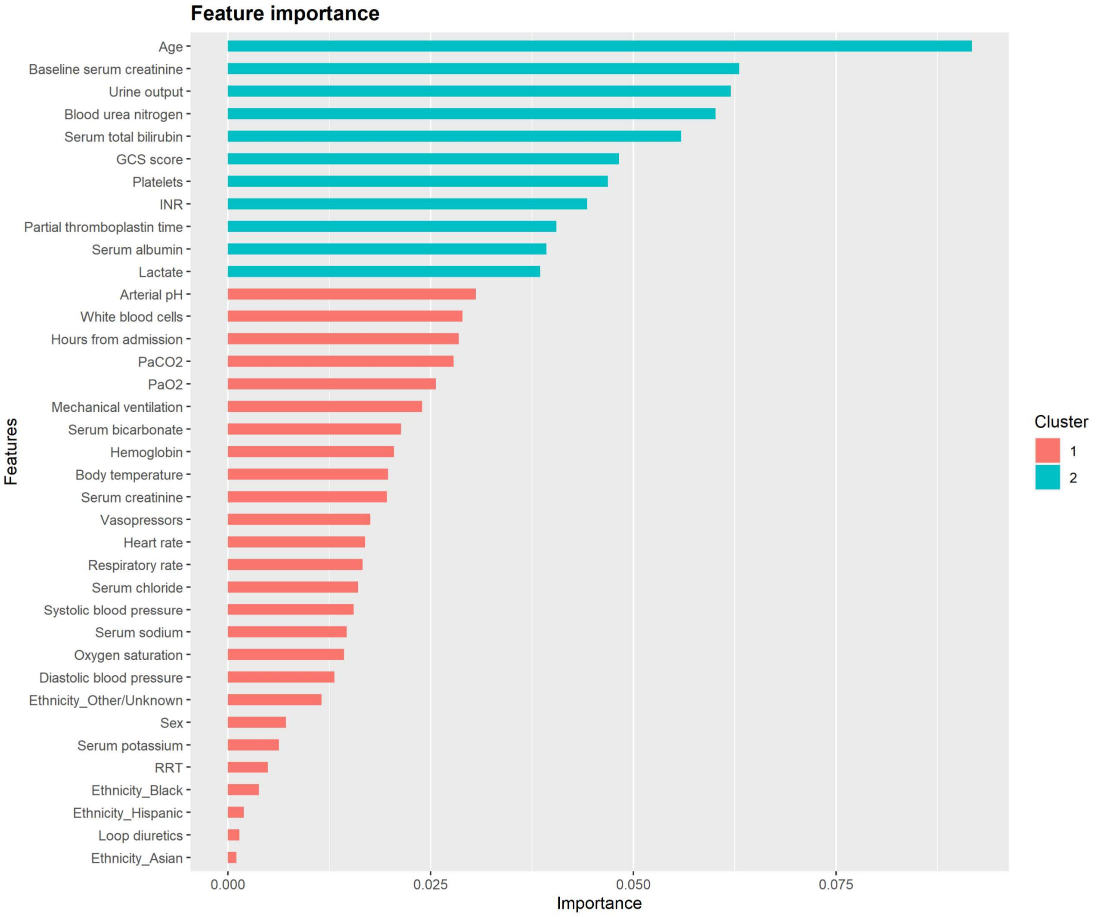

Figure S10. Feature importance derived from the XGBoost model for mortality in the first 28 days. GCS, Glasgow Coma Scale; INR, international normalized ratio; PaCO2, partial pressure of arterial carbon dioxide; PaO2, partial pressure of arterial oxygen; RRT, renal replacement therapy.

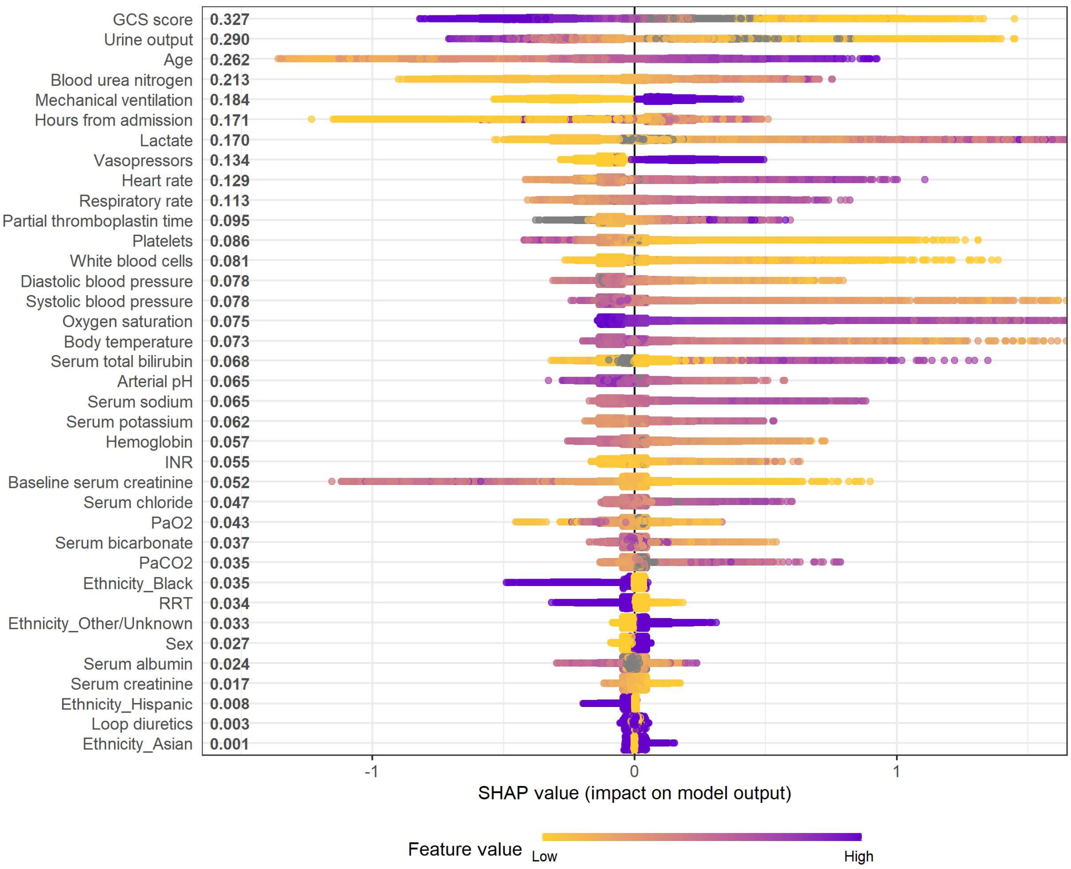

Figure S11. SHAP summary plot of the XGBoost model for mortality in the following 72 hours. GCS, Glasgow Coma Scale; INR, international normalized ratio; PaO_2, partial pressure of arterial oxygen; $PaCO_2$, partial pressure of arterial carbon dioxide; RRT, renal replacement therapy.

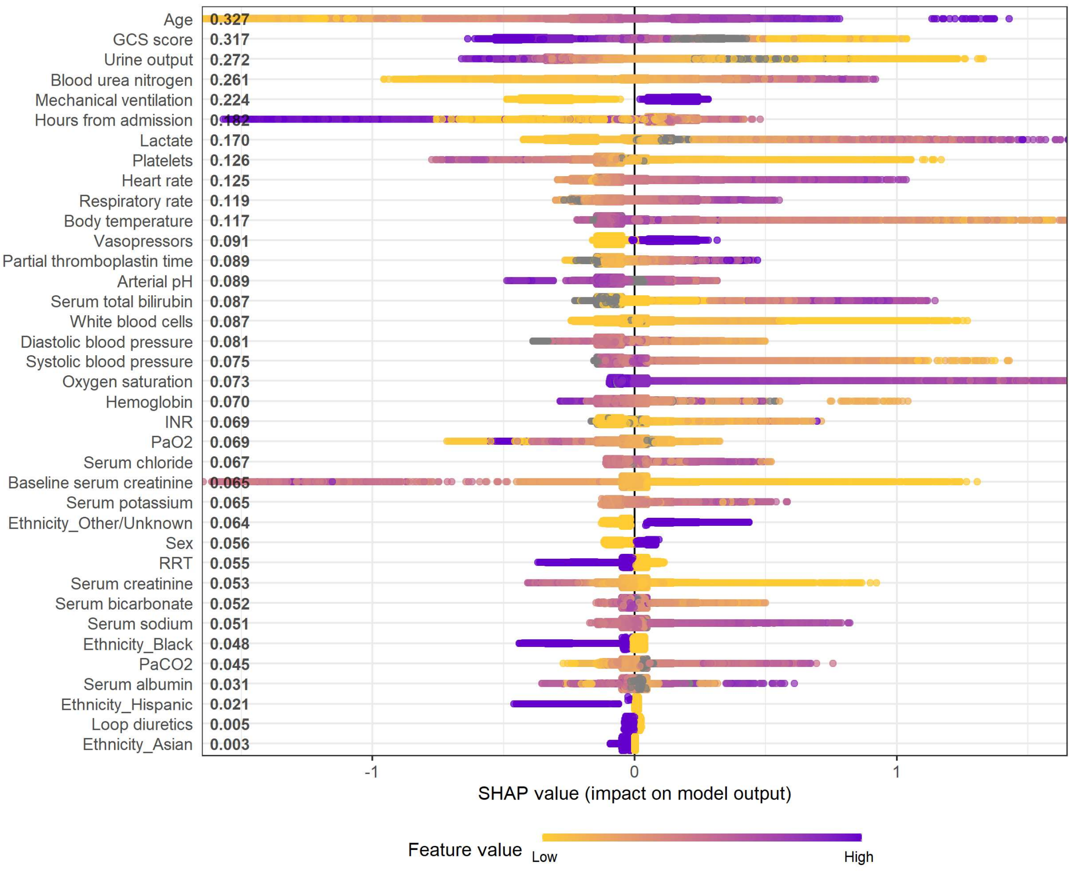

Figure S12. SHAP summary plot of the XGBoost model for mortality in the following 120 hours. GCS, Glasgow Coma Scale; INR, international normalized ratio; PaO_2, partial pressure of arterial oxygen; RRT, renal replacement therapy; $PaCO_2$, partial pressure of arterial carbon dioxide.

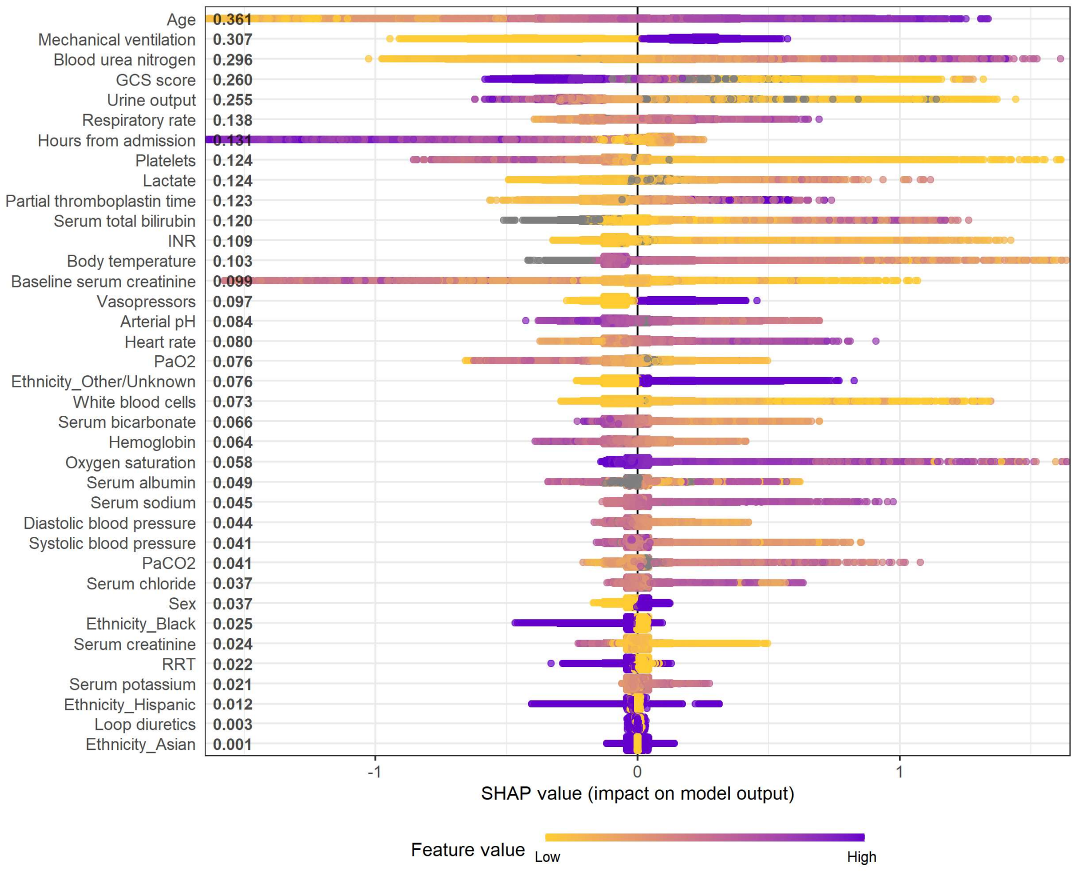

Figure S13. SHAP summary plot of the XGBoost model for mortality in the first 28 days. GCS, Glasgow Coma Scale; INR, international normalized ratio; PaO_2, partial pressure of arterial oxygen; $PaCO_2$, partial pressure of arterial carbon dioxide; RRT, renal replacement therapy.

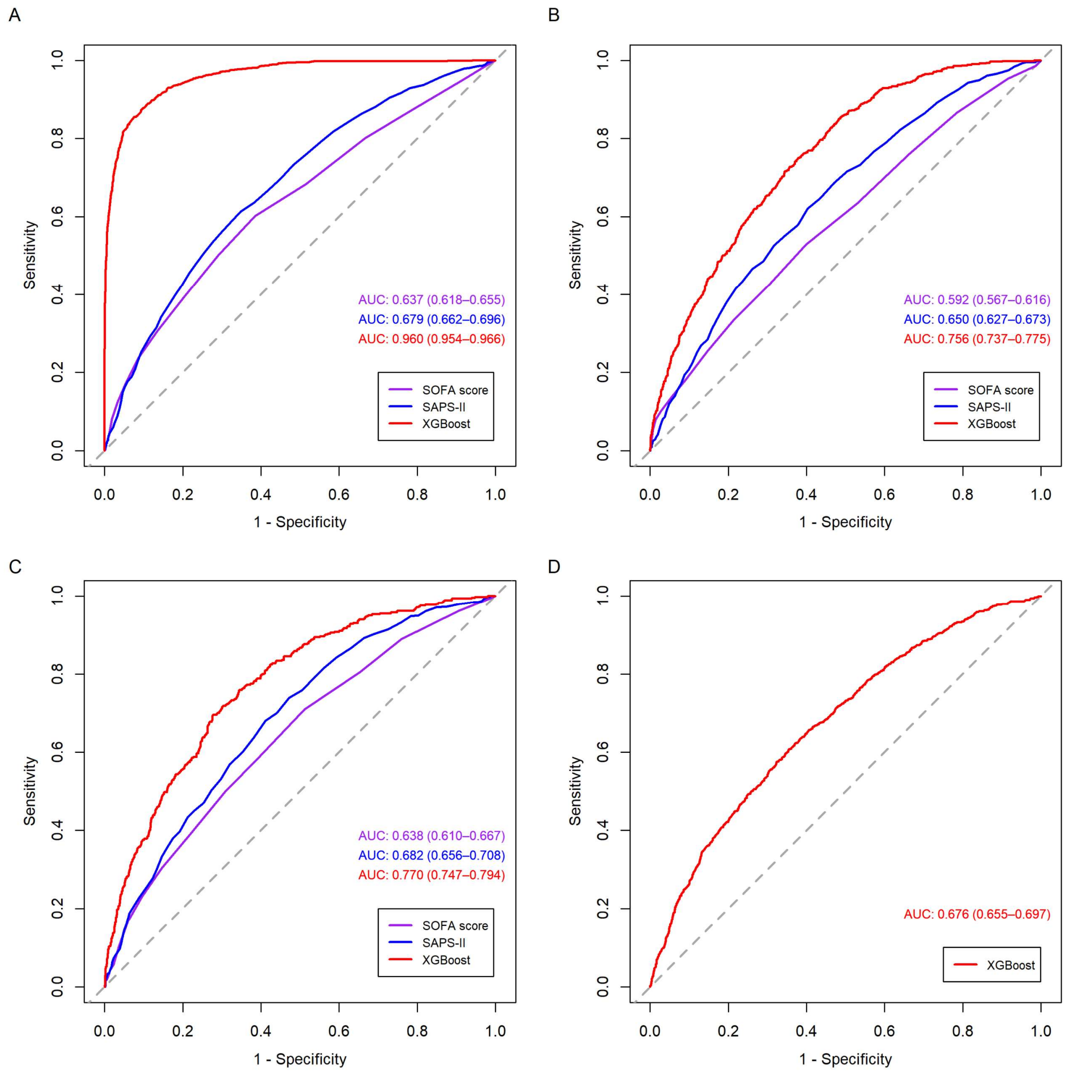

Figure S14. Receiver operating characteristic curves of the models for mortality in the first 28 days in the training set (A), validation set (B), internal test set (C) and external test set (D), using data gathered during the first 12 hours after ICU admission. SOFA, Sequential Organ Failure Assessment; SAPS-II, Simplified Acute Physiology Score II; XGBoost, eXtreme Gradient Boosting.
